# Low-resolution structural studies of human Stanniocalcin-1

**DOI:** 10.1186/1472-6807-9-57

**Published:** 2009-08-27

**Authors:** Daniel M Trindade, Júlio C Silva, Margareth S Navarro, Iris CL Torriani, Jörg Kobarg

**Affiliations:** 1 Centro de Biologia Molecular Estrutural (CEBIME), Campinas, SP, Brazil; 2 Instituto de Biologia, Departamento de Bioquímica, Universidade Estadual de Campinas, Campinas, SP, Brazil; 3 Instituto de Física "Gleb Wataghin", Universidade Estadual de Campinas, Campinas, SP, Brazil; 4 Laboratório Nacional de Luz Síncrotron (LNLS), Campinas, SP, Brazil

## Abstract

**Background:**

Stanniocalcins (STCs) represent small glycoprotein hormones, found in all vertebrates, which have been functionally implicated in Calcium homeostasis. However, recent data from mammalian systems indicated that they may be also involved in embryogenesis, tumorigenesis and in the context of the latter especially in angiogenesis. Human STC1 is a 247 amino acids protein with a predicted molecular mass of 27 kDa, but preliminary data suggested its di- or multimerization. The latter in conjunction with alternative splicing and/or post-translational modification gives rise to forms described as STC_50 _and "big STC", which molecular weights range from 56 to 135 kDa.

**Results:**

In this study we performed a biochemical and structural analysis of STC1 with the aim of obtaining low resolution structural information about the human STC1, since structural information in this protein family is scarce. We expressed STC1 in both *E. coli *and insect cells using the baculo virus system with a C-terminal 6 × His fusion tag. From the latter we obtained reasonable amounts of soluble protein. Circular dichroism analysis showed STC1 as a well structured protein with 52% of alpha-helical content. Mass spectroscopy analysis of the recombinant protein allowed to assign the five intramolecular disulfide bridges as well as the dimerization Cys202, thereby confirming the conservation of the disulfide pattern previously described for fish STC1. SAXS data also clearly demonstrated that STC1 adopts a dimeric, slightly elongated structure in solution.

**Conclusion:**

Our data reveal the first low resolution, structural information for human STC1. Theoretical predictions and circular dichroism spectroscopy both suggested that STC1 has a high content of alpha-helices and SAXS experiments revealed that STC1 is a dimer of slightly elongated shape in solution. The dimerization was confirmed by mass spectrometry as was the highly conserved disulfide pattern, which is identical to that found in fish STC1.

## Background

Stanniocalcins (STCs) represent a small family of secreted glycoprotein hormones consisting of STC1 and STC2 in which amino acid sequences are highly conserved among aquatic and terrestrial vertebrates [1-7]. However, the lack of homology with other known proteins has hampered the understanding of their functions. Initial evidence suggested that mammalian STC1 would parallel the function of fish STC1, which has been implicated in mineral homeostasis [8-10]. It is tempting to assume that the functions of STC1 and STC2 overlap at least in part, since they share high similarity in their primary amino acid sequence especially at the N-terminus and the pattern of cysteine residues is highly conserved [11].

However, there are also several differences between STC1 and STC2, including the fact that STC2 has 55 additional amino acids, the majority of which are located at its C-terminus [12-14]. Furthermore their expression patterns are different[1,14-17] and STC2 is unable to displace STC1 from its putative receptor [18,19], indicating that both molecules may have distinct receptors.

Although STC1 functions as an anti-hypercalcemic hormone in fish [20-22], it is becoming increasingly clearer that it may have expanded roles in mammals. Such assumption is based on its wide expression pattern in adult normal tissues[1,16,23-27], tumors [17,28,29] and also during embryogenesis [30-35]. Further support for a complex function of STC1 in mammals comes from studies that show its varying sub-cellular localizations [18,19] and of a gain-of-function phenotype observed in transgenic mouse [36,37].

Relatively little is also known about STCs molecular structure. The human and mouse genomes encode a 247 amino acid STC1 protein [17,38]. The first 204 amino acids show 92% sequence similarity to salmon STC1 and include a conserved N-linked glycosylation site of the type Asn-X-Thr/Ser (N-X-T/S) [17,39]. Compared to the fish STC1 however, the last 43 residues at the C-terminus are poorly conserved in human STC1 (and STC2), suggesting that the main biological activity of the STCs is mediated through its N-terminus [40,41].

In ancient fish, the last conserved cysteine residue in the C-terminal of STC1, which is supposedly involved in its dimerization, is replaced by arginine or histidine residues, thereby giving rise to a strictly monomeric form of the protein [42,43]. Although dimeric forms of STC1 have been described [39,44,45], answers to the question of its potential multimerization and modification to diverse higher molecular weight forms under certain circumstances remain elusive.

STC1 however, seems to exist in two different forms, the conventional dimeric 56 kDa form, consisting of two ~28 kDa monomers, also known as STC_50_, and a number of higher molecular weight STC variants, collectively referred to as "big STC" [19,25,46-49]. At least three molecular weights: 84, 112, and 135 kDa have been described and big STC1 has been reported to be expressed in adipocytes, adrenocortical cells [47,48] and ovaries [19,25,49]. In order to explain the increased mass of big STC1 it has been suggested that either distinct post-translational modifications, including glycosylation [17,25] or phosphorylation [50] occur in big STC or additional but yet uncharacterized exons [48] are being employed. In agreement with the latter, the monomeric form big STC1 is about ~10 kDa larger than the theoretically predicted monomer. Another possibility is the formation of tri- (84 kDa), tetra- (112 kDa) or even pentamers (140 kDa) of STC1, although in this case obtained values only add up for the theoretically predicted monomer (~28 kDa) but not for that observed for the big STC monomer (~38 kDa). It is however noteworthy that the 135-kDa variant of big STC1 found in adrenocortical cells is resistant to chemical reduction, just like STC_50 _from the mitochondrial matrix [48], thereby suggesting the formation of a more stable and maybe durable quaternary structure.

In this paper we present structural information about the human STC1 protein. We expressed human STC1 in insect cells using a bi-cistronic baculovirus construct. After affinity purification we collected SAXS data for STC1 in solution. Data analyses are indicative of a dimeric protein in solution. Furthermore, we were able to confirm the formation of the conserved disulfide bridges, previously reported in fish STC1, by mass spectrometry.

## Results and Discussion

### STC1 is predicted to be dimer and to possess a high content of alpha-helices

**Figure 1:**
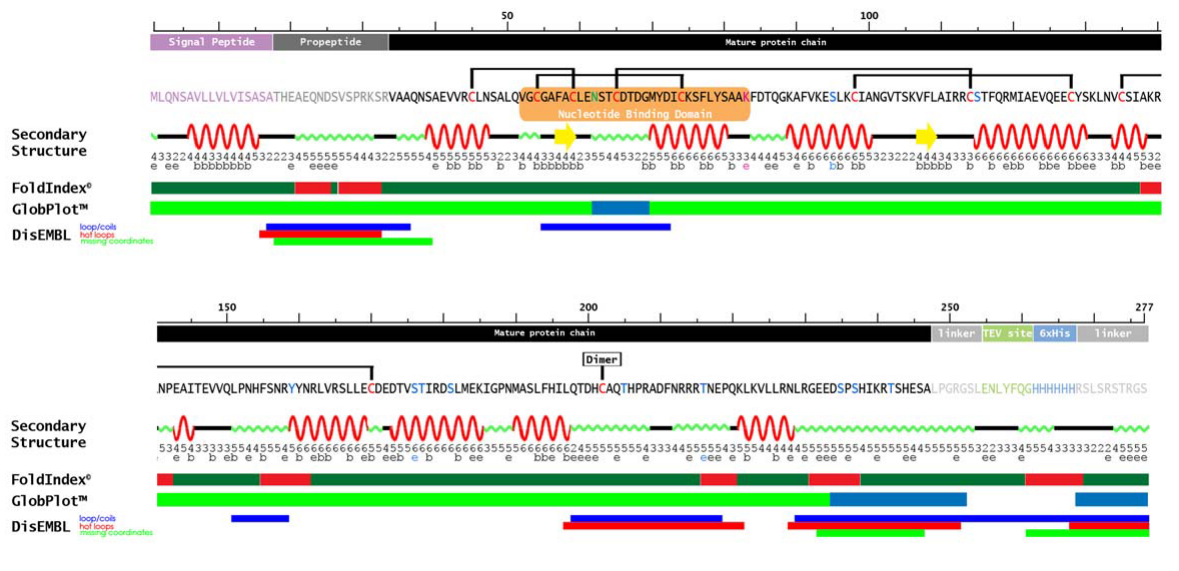
**Prediction of secondary structure and putative post-translational modification sites in the human STC1 amino acid sequence**. Linear representation of STC1-HT amino acid sequence with assignment of its different regions from N- to C-terminus: signal peptide (purple), pro-peptide (dark gray), mature protein (black), linker regions (light grey), TEV protease cleavage site (green) and 6 × His-tag (light blue). In the amino acid sequence, relevant residues are emphasized by the following color code: Cys: red, Asp predicted to be N-glycosylated: green, Lys predicted to be sumoylated: magenta, Ser, Thr e Tyr residues predicted to be phosphorylated: blue. The conserved pattern of experimentally determined disulfide bridges from salmon STC1 is indicated by black horizontal brackets. Similarly the homo-dimerization Cys is indicated in black (dimer). Below the sequence there is a schematic representation of the predicted consensus secondary structure, obtained by six different prediction programs (red: alpha helix, yellow: beta-sheet, green: coil regions, black: not assigned). The numbers below the secondary structure represent the score (1-6, indicating how many of the six programs predicted the respective secondary structure element). Furthermore, in a second line, a prediction indicates whether a residue is exposed (e) or buried (b). At the bottom, predictions of three programs for ordered/disordered regions are given: FoldIndex (red: unfolded, green: folded), GlobPlot (green: globular, blue: disordered) and DisEMBL (blue: loops or coils, red: hot loops, green: missing coordinates).

By analysing the human STC1 amino acid sequence using six different secondary structure prediction databases, we created a secondary structure consensus and scored it by the number of times (one to six times) the predicted secondary structure element scored positive (Figure 1). Prediction programs used were: **PredictProtein/PROF **[51], **PsiPRED **[52], **Predator **[53], **SOPMA **[54], **SSPro **[55] and **JCFO **[56].

In summary, the secondary structure analysis suggested that about 34% of the amino acid sequence of STC1 may form alpha-helices.

We further performed some predictions about ordered or disordered regions within the sequence using **FoldIndex **[57] and **DisEMBL **[58]) as well as **GlobPlot **[59] as a predictor for more globular regions (Figure 1). The first two programs both predicted that the pro-peptide region and possibly the C-terminal region, this last one which contains the Cys disulfide mediated dimerization region, to be highly disorder or a region with high loop/turn content.

We analyzed and plotted (Figure 1) the conserved cysteine residues as well as the experimentally determined disulfide bridges from the salmon sequence determination [39], the signal peptide, pro-peptide and mature protein sequence as annotated at UniProtKB/Swiss-Prot database (Swiss-Prot:P52823), and we also emphasize a previously described nucleotide binding domain (NBD) [60].

Additionally, some predictions about post-translational modifications were performed and compared to published experimental data. An N-glycosylation site which had already been characterized for STC1 [17,41,61] was also predicted by **NetGlyc **[62] (Figure 1)

For phosphorylation analysis we combined prediction data from **NetPhos **[63] and **NetPhosK **[64] together with *in vitro *phosphorylation data [50] to annotate tyrosine, threonine and serine residues as putative phosphorylation sites (Table 1). Most of the kinases that were found to phosphorylate STC1 by the in vitro phophorylation screening of Jellinek and coworkers were predicted by both prediction programs (Table 1), except calmodulin-dependent protein kinase (CaMPK-II) and casein kinase II (CK2).

**Table 1:** Prediction of putative post-translational modification sites in human STC1.

**Residue**	**Modification**	**Buried/Exposed** **Residue**[51]	**Predictor (Score)**	**Ref**.
N_62_	N-glycosylation	nd	NetGlyc (0.61)	[17,39,41,61]

K_83_	Sumoylation	e	SUMOplot™ (0.79)	$

S_95_	PKC* phosphorylation	b	NetPhos (0.844)/NetPhosK (0.630)	[50]

S_115_	PKC* phosphorylation	nd	NetPhos (0.788)/NetPhosK (0.722)	[50]
	PKA* phosphorylation	nd	NetPhos (0.788)/NetPhosK (0.841)	[50]
	RSK* phosphorylation	nd	NetPhos (0.788)/NetPhosK (0.601)	nd

Y_159_	INSR* phosphorylation	nd	NetPhos (0.929)/NetPhosK (0.539)	nd

S_176_	PKC* phosphorylation	e	NetPhos (0.938)/NetPhosK (0.630)	[50]

T_177_	PKC* phosphorylation	nd	NetPhos (0.983)/NetPhosK (0.640)	[50]

S_181_	PKA* phosphorylation	nd	NetPhos (0.993)/NetPhosK (0.647)	[50]

T_205_	PKC* phosphorylation	nd	NetPhos (0.606)/NetPhosK (0.815)	[50]
	Cdc2* phosphorylation	nd	NetPhos (0.606)/NetPhosK (0.509)	[50]

T_216_	PKG* phosphorylation	e	NetPhos (0.817)/NetPhosK (0.600)	[50]

S_235_	GSK3* phosphorylation	nd	NetPhos (0.986)/NetPhosK (0.508)	[50]
	Cdk5* phosphorylation	nd	NetPhos (0.986)/NetPhosK (0.551)	[50]

S_237_	PKC* phosphorylation	nd	NetPhos (0.531)/NetPhosK (0.647)	[50]

T_242_	PKG* phosphorylation	nd	NetPhos (0.523)/NetPhosK (0.693)	[50]

High score predictions of glycosylation, sumoylation and phosphorylation on STC1 sequence are presented. Predicted modifications within the pro-peptide region were excluded. Putative phosphorylated residues shown here are only those that were both predicted with the highest scores by the NetPhos server and additionally were predicted by NetPhosK, which suggests a specific kinase for the same site. References are related to additional experimental support for the predicted modification, if available. * indicate kinase as predicted by NetPhosK [protein kinase A C or G (PKA; PKC and PKG); 90-kDa Ribosomal S6 Kinase (pp90RSK or RSK); Insulin receptor (INSR); cell division cycle 2 (cdc2 or p34 protein kinase); ciclin dependent kinase 5 (cdk5); Glycogen synthase kinase 3 (GSK3)]; e = exposed residue, b = buried residue, nd = not determined; $ = unpublished data.

Analysis by **PredictProtein/PHD Acc **[51] revealed that some of the residues such as S_176 _and T_216_, are predicted to be exposed to the solvent and therefore more likely to suffer phosphorylation. Indeed, both residues refer to STC1 kinase sites found to be phosphorylated by Jellinek and co-workers [50].

In order to screen for lysine residues predicted which may be sumoylated in STC1 we used SUMOplot™  and found three putative sumoylated residues (data not shown). The one having the highest score is located at the end of the NBD and the sumoylated residue (K_83_) is also predicted by **PredictProtein/PHD Acc **to be exposed to solvent. Most interestingly, we found that STC1 interacted with the SUMO1 protein in a yeast two hybrid screen (unpublished data). These data suggest that further experiments should be performed to test if sumoylation of STC1 may occur in vivo, in human cells.

### Optimization of the expression and purification of STC1

**Figure 2:**
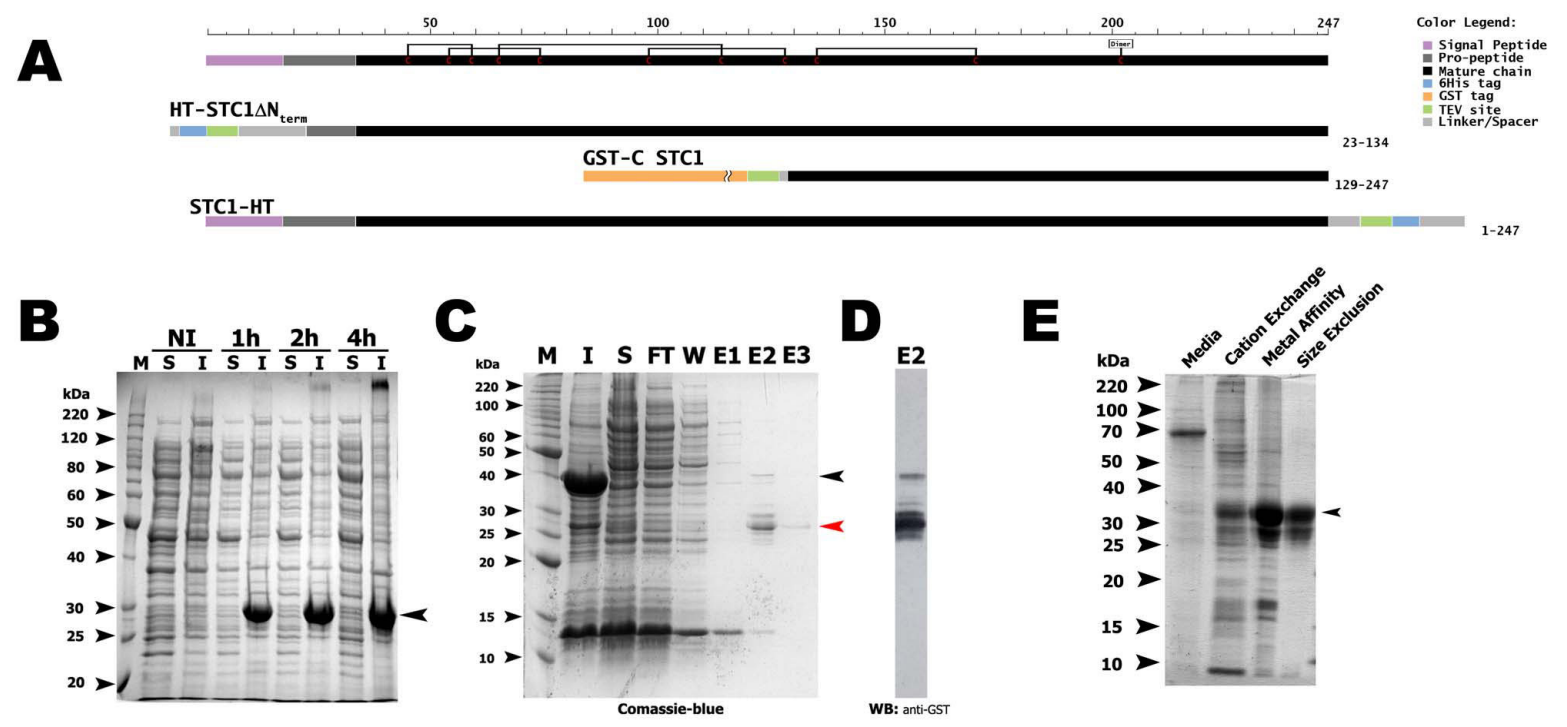
**Large scale STC1 expression in E. coli and in insect cells and its purification**. **(A) **Tested STC constructs (from top to bottom): an amino-6 × His tagged STC1 without the N-terminal portion which includes the signal peptide (HT-STC1ΔNterm), an amino-GST tagged C-terminal fragment of STC1 (GST-C STC1) and a full length carboxy-6 × His tagged STC1 (STC1-HT). At the right side of each construct is shown the amino acid residues from native STC1 present on that construct. **(B) **Expression test of HT-STC1ΔNterm. Coomassie-blue stained SDS-PAGE of soluble (S) and insoluble (I) fractions expressed in BL21DE3 non-induced (NI) or induced for indicated periods with 0.5 mM IPTG in LB at 37°C. **(C) **GST-C STC1 purification by affinity chromatography using glutathione sepharose beads. Coomassie-blue stained SDS-PAGE of insoluble (I), soluble (S), flow-through (FT), wash (W) and elution (E1-E3) fractions. **(D) **Western blot anti-GST of E2 fraction of purification shown in C. Black arrow heads at right indicate expected recombinant protein size and red arrow head indicates un-fused GST protein. **(E) **Expression and purification of STC1-HT from insect cells (using the baculo virus system): Coomassi-blue stained SDS-PAGE of peak-fractions after cation exchange, metal affinity and Size Exclusion chromatography. Arrow head indicates expected size of recombinant expressed protein. Invitrogen Bench Marker protein ladder (M).

Our first attempt to produce STC1 in *E. coli *using the HT-STC1ΔNterm construct (Figure 2A) resulted in completely insoluble expression (Figure 2B). Even splitting the protein in two halves using His-tag fusion did not make any difference in solubility, since both parts still expressed in insoluble form (data not shown). Only together with the use of GST-tag (GST-C STC1) we could obtain some soluble expression, however at very low amounts. The highest rate of soluble expression could be obtained with GST-C STC1 (Figure 2C and 2D).

On the other hand, using a modified bi-cistronic vector of the baculovirus expression system we could obtain milligrams per liter of the soluble full-length his-tagged STC1 (STC1-HT) secreted into the media (Trindade et al., unpublished data). The amount of virus and of infected cells could be easily optimized, since the recombinant bi-cistronic baculo virus promotes production of endogenous GFP protein, turning infected cells green.

Purification was obtained by a three step chromatography of the media: cation exchange followed by metal-affinity and size exclusion chromatographies (Figure 2E). Several milligrams of protein were routinely obtained per liter of culture supernatant and the obtained protein was used for subsequent experiments.

### Confirmation of disulfide bonds by mass-spectrometry

By analysing the recombinant human STC1-HT produced in the baculovirus system by ESI/Q-TOF analysis we were able to identify and assign the peptides that resulted from enzymatic digestion either with trypsin or chymotrypsin in the oxidized and/or reduced forms (Table 2, [see Additional file 1, 2 and 3]). In brief, the data show the existence of peptides having mass compatible with the presence of the previously predicted disulfide bonds for the salmon STC1. In Table 2, the first column gives the disulfide bridge in question and the last four columns give respectively the expected and experimentally determined peptide masses. In conclusion, all disulfide bridges except for one could be directly demonstrated. Still Cys_45_-Cys_59 _could be evidenced indirectly, since the mass of the peptide shown in line one of Table 2 is compatible with this interpretation. Furthermore, after chemical digestion with formic acid, Cys_202 _could be unambiguously assigned as the Cys residue responsible for the dimerization of human STC1 (Table 2, [see Additional file 1, 2 and 3]).

**Table 2:** Identification of signature peptide sequences of STC1-HT for the assignment of the intra- and intermolecular disulfide bonds.

			**Mass**			
			
**Disulfide Bond**	**Sequence of peptides**	**Protease**	**Theoretical**	**Observed (Expected)**		
				
				**[M+2H]**	**[M+3H]**	**[M+4H]**
C_45_-C_59_; C_54_-C_74_; C_65_-C_114_	**C**_45_LNSAL...ID**C**K_75_ **C**_114_STFQR_119_	Trypsin	3981.51			996.22 (996.38)

C_54_-C_74_	Q_51_VG**C**GAFA_57_ D_72_I**C**KSF_77_	Chymotrypsin	1389.61	695.95 (695.81)	464.24 (464.21)	

C_65_-C_114_	E_61_NST**C**...GMY_71_ A_110_IRR**C**STF_117_	Chymotrypsin	2184.89		729.36 (729.30)	547.26 (547.23)

C_98_-C_128_	**C**_98_IANGVTSK_106_ M_120_IAE...**C**YSK_131_	Trypsin	2318.06		773.73 (773.69)	580.56 (580.52)

C_98_-C_128_	K_97_**C**IA...SKVF_108_ Q_118_RMIA...EE**C**Y_129_	Chymotrypsin	2761.32		921.17 (921.45)	691.14 (691.34)

C_135_-C_170_	L_132_NV**C**SIAK_139_ S_166_LLE**C**...TIR_179_	Trypsin	2424.19		809.12 (809.07)	607.08 (607.05)

			**Mass**			
			
**Disulfide Bond**	**Sequence of peptides**	**Chemical Reagent**	**Theoretical**	**Observed (Expected)**		
				
				**[M+H]**		

C_202_-C_202_*	D_200_H**C**AQTHPRA_209_ D_200_H**C**AQTHPRA_209_	Formic acid	2266.98	2268.11 (2267.99)		

Samples were digested by trypsin or chymotrypsin with or without dithiotreitol and iodoacetamide, separated by UPLC and analyzed by ESI-QTOF; or digested with formic acid and analyzed by MALDI-QTOF. The presented mass is the monoisotopic. Dimer disulphide bond is indicated by asterisk (*) [see Additional file 1, 2 and 3].

### Analysis of secondary structure

**Figure 3:**
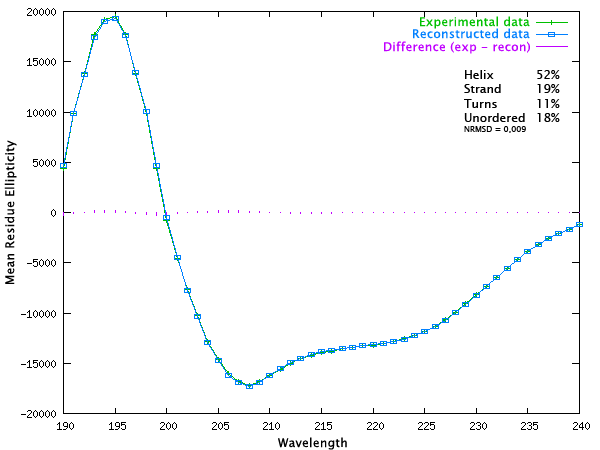
**Circular Dichroism spectra of STC1-HT and deconvolution**. Graph of the wavelength plotted against the mean residue elipticity of a sample at 5,5 mM in 10 mM MES; 33,3 mM NaCl pH 6,5 at 4°C. Data were deconvoluted with the CDSSTR program on the Dichroweb server. Note the two minima at 208 and 222 nm, which are typical of alpha-helix containing proteins. Reconstructed data are those derived from the Dichroweb database.

Such a relatively high content predicted by *in silico *analysis (Figure 1) is supposed to be readily detected by circular dichroism spectroscopy of the protein, so the content of secondary structure elements in recombinant human STC1-HT was determined by circular dichroism spectroscopy. Figure 3 shows the spectrum of purified STC1 recorded at 4°C. Purified protein presents negative ellipticity in the near-UV, with minima at 208 (-17.2 × 10^3 ^deg cm^2 ^dmol^-1^) and 222 nm (-12.8 × 10^3 ^deg cm^2 ^dmol^-1^). Deconvolution of the CD spectrum lead to the following estimation of the content of secondary structural elements: ~52% of α-helices, ~19% of β-sheets strands, ~11% of turns and ~18% unordered (NRMSD = 0,009) using the CDSSTR algorithm on the Dichroweb web server [65]. Consensus predictions of secondary structures shown in Figure 1 give values of about 37% of helix, 2.5% of strands and 65.5% of other structures (37% of coils and 28.5% of non-determined). Secondary structural predictors like PSIPRED are based on neural networks trained on known folds, and thus tend routinely to underestimation of the true helical and strand content, due to the fact that the reference databases are not complete. A more critical issue is the fact that no other protein of the family of STCs has its structure resolved. In conclusion both the prediction and the experimentally determined data are in reasonable agreement, since they demonstrate a relatively high content of alpha-helices in human STC1.

### STC1 is a compact, slightly ellipsoidal dimer in solution

Dynamic Light Scattering (DLS) data of the recombinant STC1 sample showed a single and narrow peak, which is an indicative of a monodisperse solution of dimers.

**Figure 4:**
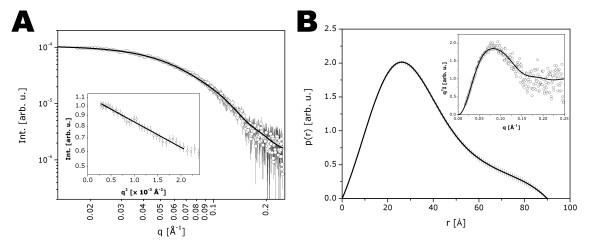
**Experimental Small Angle X-ray Scattering (SAXS) curves for recombinant STC1-HT protein**. (A) Experimental scattering curve of STC1-HT (open circles) and the theoretical fitting (solid line) by using the program GNOM. *Inset*: Guinier Region. (B) Pair-distance distribution function p(r). *Inset*: Kratky representation of the intensity curve.

The corrected and normalized experimental SAXS data are shown in Figure 4A, together with the GNOM curve fitting. The Guinier region providing an Rg value of 27.4 ± 0.8 Å is shown in the inset. The p(r) function resulting from these calculations is shown in Figure 4B, with an inset showing the Kratky representation of the intensity curve. The Kratky plot indicates a slightly compact conformation for STC1 in solution. The maximum dimension (D_max_) value obtained was 90 Å and the Rg value, calculated from the p(r) function, was 27.8 ± 0.4 Å, in close agreement with that calculated from the Guinier approximation. As it can be noted from the p(r) function shape, STC1 has a slightly elongated shape.

Using BSA as a reference sample, the molecular mass for STC1 HT, estimated from the SAXS data, was ~54 kDa. This value is in agreement with the prediction of the protein being a dimer, since the theoretically calculated molecular mass of the monomer was ~27 kDa (calculated from the amino acids sequence using ProtParam tool [66]).

The dimerization was also confirmed both by mass spectrometry (see above) and by size exclusion chromatography (data not shown).

### Low resolution *ab initio *SAXS-based models for STC1

**Figure 5:**
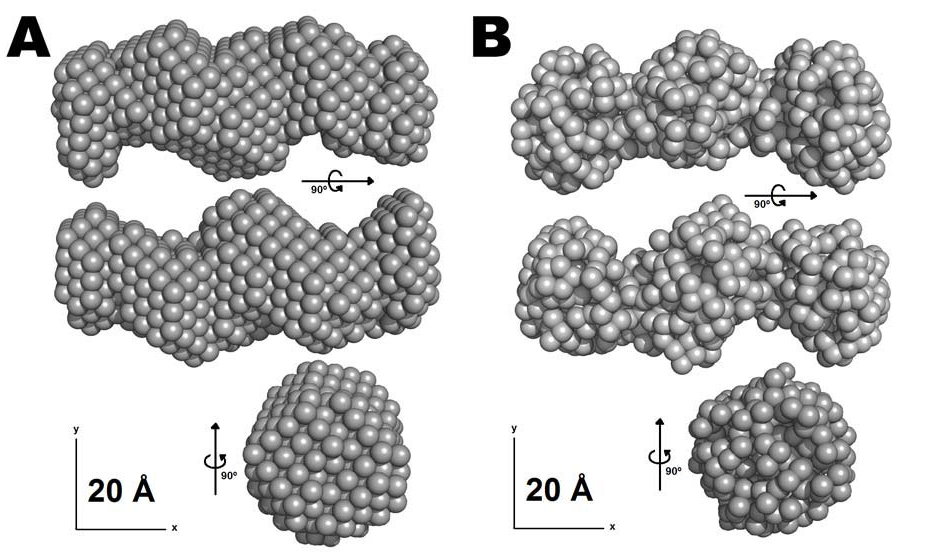
**Low resolution *ab initio *model of STC1-HT derived from SAXS data**. (A) Three selected views of the average and filtered *dummy atoms *model (DAMMIN). (B) Three selected views of the *dummy residues *model (GASBOR). The models were displayed by the PyMOL program [80].

The low resolution models for STC1 are presented in figure 5. Those models were derived from the experimental SAXS data imposing a 2 point symmetry constraint (P2). Additional models calculated without symmetry constraint (P1) presented very similar molecular envelopes. The calculated values of the Normalized Spatial Discrepancy (NSD), which is an indicator of the difference between models, gave values of ~0.6 for P1 vs. P2 DAMMIN models and ~0.8 for P1 vs. P2 GASBOR models, suggesting a low discrepancy. In view of this result, all model calculations were performed using a 2 point symmetry constraint. After several runs performed with the program DAMMIN, the averaged and filtered (with the corrected excluded volume) *dummy atom *model for STC1 is shown in Figure 5A. The NSD values for the set of 10 models ranged from 0.60 to 0.69, which are considered reasonable values [67]. This low resolution model shows the expected elongated shape for the protein dimer. The most typical and recurrent *dummy residue *model resulting from the calculation with the program GASBOR is shown in Figure 5B. The NSD values for this set of 10 calculations ranged from 0.82 to 0.87, which are also quite reasonable. This last approach produced an improved molecular envelope for STC1. Comparing the results, both molecular envelopes obtained for STC1 presented a similar shape and confirmed the elongated conformation for the dimer.

## Conclusion

Our data provided the first low resolution 3D structure of human STC1 protein in solution. SAXS experiments indicated that STC1 forms a dimer of slightly elongated shape in solution. Circular dichroism spectroscopy confirmed the prediction of a high alpha-helical content and we could also confirm by mass spectrometry the highly conserved disulfide pattern, previously described in fish STC1[39]. Disulfide bonds are formed between the same 10 of the 11 conserved Cys, in the same fashion, leaving the C-terminal Cys 202 free to engage in dimer formation. None of our data explain the composition or structure of "bigSTC1" previously reported to appear in certain tissues [19,25,46-49]. Indeed, our results only show the formation of dimers (STC_50_), by several independent methods. In human cells however, we may have additional contributions from possible post-translational modifications or alternative splice variants of the pre-mRNA encoding STC1, which may contribute to the appearance of the higher molecular weight forms. Further experiments are required to characterize big STC1 at the molecular level and point out its differences with the canonical dimeric human STC1.

## Methods

### *In silico *sequence analysis

We analyzed the human STC1 sequence as a query in six different secondary structure prediction databases (PredictProtein/PROF [51], PsiPRED [52], Predator [53], SOPMA [54], SSPro [55] and JCFO [56]). We also performed some predictions about ordered or disordered regions within the sequence using FoldIndex [57] and DisEMBL [58]) as well as GlobPlot [59], a predictor for globular regions. Additionally, some predictions about post-translational modifications were done and compared to previous published data. N-glycosylation sites were predicted by NetGlyc [62]. For phosphorylation we combined prediction data from NetPhos [63] and NetPhosK [64]. With PredictProtein/PHD Acc [51], we predicted whether residues are exposed to solvent or buried. Finally, in order to screen for lysine residues predicted which may be sumoylated in STC1 we used SUMOplot™ .

### Cloning of STC1 cDNA

Full-length STC1 (Genbank NM_003155) gene was amplified from normal bone marrow stromal cells using primers STC1 F (5' aaggatccAGAATGCTCCAAAACTCAGC 3') and STC1 R (5' ccgaattCCTCTCCCTGGTTATGCAC 3') and cloned into vector pGEM resulting in plasmid **pGEM-STC1**. In order to obtain all constructs we used pGEM-STC1 as template and cloned PCR amplified products into pGEM plasmid: for **pGEM-STC1 ΔN_term_**(STC1 lacking the first 22 amino acids) we used primers STC1 ΔN_term _F (5' aaggatccCAGAATGACTCTGTGAGCCC 3') and STC1 R; for **pGEM-STC1 full without stop **(STC1 without stop-codon) we used primers STC1 F and STC1 no stop R (5' acaagcttCCTCTCCCTGGTaATGCAC 3'); for **pGEM-CSTC1 **(C terminal of STC1 consisting of residues from 129 to 247) were used primers CSTC1 F (5' ggatccTACAGCAAGCTGAATGTGTG 3') and CSTC1 R (5' gaattcTTATGCACTCTCATGGGATG 3'). Capital letters indicate sequence identical to STC1 cDNA, small caps letters indicate sequence non-identical to template. All pGEM constructs were verified by DNA sequencing in order to ascertain the correct nucleotide sequence. pGEM-STC1 ΔN_term _and pGEM-CSTC1 were digested with BamHI and EcoRI and the resulting inserts were cloned into pET28a-His-Tev or pET28a-GST-Tev [68] previously digested with the same endonucleases. This resulted in pET-HT-STC1 ΔN_term_, pET-HT-CSTC1, and the pET-GST-CSTC1 constructs. pGEM-STC1 full without stop was digested with BamHI and HindIII and cloned into a pFastBAC Dual+EGFP (pFBDg), which had the EGFP cDNA cloned under p10 promoter, digested with same endonucleases to insert STC1 under polyhedron promoter. Subsequently a pair of oligonucleotides (5'AGCTTGGAAAACCTGTATTTTCAGGGCCATCACCATCACCATCACCGG 3' and 5'AGCTCCGGTGATGGTGATGGTGATCGCCCTGAAAATACAGGTTTTCCA 3') previously annealed was added to generate a linker consisting of a TEV protease site and a 6 × His-tag (HT) at the C-terminal, resulting in the pFBDg-STC1-HT construct. Other constructs mentioned in the text were generated by using the same methodology.

### Expression and purification of STC1

Production of the recombinant 6 × His- or GST-STC1 fusion constructs in *E. coli *BL21 strain and subsequent purification trials were performed as described previously for other recombinant proteins [69,70].

High Five™ (Invitrogen) cells were adapted to grow in suspension culture in Express Five™ serum free media (Gibco) supplemented with 20 mM L-Glutamine (Gibco) and 1× PenStrep (Gibco). The stock cell culture was maintained and passaged in a 28°C incubator (ThermoForma). For STC1-HT production High Five cells were scaled up from the stock culture to a cell density of 1 × 10^6 ^in two 2L Erlenmeyer flasks containing 500 mL each and incubated at a shaker at 26°C at 140 rpm. Twelve hours post inoculation, the cells were infected with the recombinant baculo virus, at a multiplicity of infection (m.o.i.) between 3 and 4 plaque-forming unit (pfu) per cell. Baculovirus-infected High Five culture media were harvested after 48 hours post-infection by centrifugation at 500 × g for five minutes and cell-free supernatant containing secreted STC1-HT was used for purification. To the baculovirus supernatant a 1 M MES stock solution was added to bring the solution to a final concentration of 50 mM MES pH 6.5 (IEX buffer). The solution was filtered through a 0.45 μm MCE membrane (Fisherbrand) and loaded onto a water-jacketed chilled (4°C) XK26/20 (Pharmacia Biotech/GE) column previously packed with SP Sepharose FF (Pharmacia Biotech/GE) at a flow rate of 1 mL/min using a peristaltic pump (Biologic LP - Biorad). Column was transferred to an ÄKTA FPLC system (GE) for protein elution using a 0-1 M gradient of NaCl in IEX buffer. Fractions eluted from a conductivity of 30 mS/cm onward, contained most of stanniocalcin 1 protein and were pooled. This pool was directly loaded onto a pre-packed HisTrap crude FF 5 mL (GE) column, equilibrated with 50 mM MES pH 6.5, 500 mM NaCl (affinity buffer). After injection of sample the column was washed with six column volume (CV) of affinity buffer, with three CV of affinity buffer containing 250 mM Imidazole and finally with four CV of affinity buffer containing 1 M Imidazole. This last pool of fractions containing most of stanniocalcin was concentrated using an Amicon Ultra-15 Centrifugal Filter Unit with Ultracel-10 membrane of 5,000 NMWL (Millipore) on a swing-rotor at 4°C and then 500 μL applied to a water-jacketed chilled (4°C) Superdex 200 pg 16/60 (GE) column, pre-equilibrated with 60 mM MES 600 mM NaCl pH 6.5 (SizeEx buffer) with a flow rate of 0.5 mL/min. Protein eluted at a single peak between 70 and 80 mL was analyzed by SDS-PAGE, pooled, concentrated and kept in SizeEx buffer at 4°C. The purity of the recombinant STC1 protein was confirmed by mass spectrometry analysis, which resulted in the exclusive identification of STC1 peptides (data not shown).

### Disulfide bond and molecular mass analysis

Samples digested by trypsin or chymotrypsin, treated or not with dithiotreitol and iodoacetamide, were analyzed by using ultra-performance liquid chromatography (UPLC NanoAcquity, Waters) coupled with eletrospray ionization quadrupole time-of-flight tandem mass spectrometer (ESI-QTOF Ultima, Waters/Micromass). Samples chemically digested by formic acid [71], treated or not with dithiotreitol and iodoacetamide, were analyzed using MALDI-QTOF (Q-Tof Premier, Waters/Micromass). Data were analyzed by the MassLynx software package.

### Circular Dischroism

Circular dichroism spectra were recorded at 4°C between 190 and 260 nm on a J-810 Jasco spectropolarimeter equipped with a Peltier-type system PFD 425S using a quartz cuvette of 10 mm path length, with a 50 nm/min scanning speed and a band-width of 0.5 nm. Twenty spectra of purified STC1-HT at 2.77 μM in dilution buffer (10 mM MES 33.3 mM NaCl pH6.5) were averaged and corrected from the baseline for buffer solvent contribution. Experimental data were analyzed using CDSSTR on Dychroweb web server [65].

### Small Angle X-Ray Scattering and Analysis

Before the analysis, the sample was inspected by dynamic light scattering (DLS) to test the monodispersity of the solution. After that, the sample was centrifuged at 20.000 × g for 30 min at 4°C to remove any possible aggregates. The small-angle X-ray scattering experiments were performed at the D02A-SAXS2 beam line at LNLS. The measurements were performed at 4°C under temperature-controlled conditions (via water circulation) using a 1 mm path length cell with mica windows and a monochromatic X-ray beam (wavelength of λ = 1.488 Å). The X-ray patterns were recorded using a two-dimensional position-sensitive MARCCD detector and a sample-to-detector distance of 902 mm, resulting in a useful scattering vector range of 0.015Å^-1 ^< q < 0.25 Å^-1^, where q is the magnitude of the **q**-vector defined by q = (4π/λ)sinθ (2θ is the scattering angle). Three successive frames of 300 seconds each and one frame of 30 minutes were recorded. The measurements were performed with two different concentrations for the sample in MES buffer (60 mM MES 200 mM NaCl pH 6.5): 0.15 and 0.18 mg/mL, both measured using the BCA™ Protein Assay Kit (Pierce). The buffer scattering curves were recorded keeping the same conditions used for the sample. The intensity curves were individually corrected for detector response and scaled by the incident beam intensity and sample absorption. Subsequently, buffer scattering was subtracted from the corresponding sample scattering. The resulting curves were inspected for radiation-induced damage, but no such effect was observed. After scaling the curves for concentration, no concentration effect was observed. A 10 mg/ml BSA (66 kDa) solution in the same sample buffer was used as molecular mass standard sample to estimated the molecular mass of STC1-HT. This value was inferred from the ratio of the extrapolated values of the intensity at the origin, I(0), from both sample and BSA solutions scattering [72,73].

The first analysis was the evaluation of the radius of gyration (Rg) using the Guinier approximation:  for qR_g _< 1 [74-76]. The Rg was also calculated from the pair distance distribution function, p(r), which was obtained by indirect Fourier transform of the intensity curve using the program GNOM [77]. The p(r) function also provided the maximum dimension (D_max_) of the molecule, Moreover, a Kratky representation [75,76] of the intensity curve (q^2^I(q) vs. q) was used to analyze the compactness of the protein conformation.

### *Ab initio *SAXS-based modeling

The low resolution models for STC1 were restored from the SAXS intensity curves using two different approaches. In the first one, implemented by the program DAMMIN [78], the protein was represented as an assembly of densely packed spherical beads (*dummy atoms*). Using simulated annealing, the program starts from a random configuration of beads and searches for a configuration that best fits the experimental pattern. Ten calculations were performed and the normalized spatial discrepancies (NSD) [67] values among them were evaluated using the DAMAVER suite. When the NSD values are not so different, an averaged and filtered model structure (with the correct excluded volume) emerges from this calculation. The second approach, in which generally a better model is obtained, was implemented using the program GASBOR [79]. In this approach, the protein is represented as a chain of *dummy residues *(DRs). The number of DRs is usually known *a priori *from the protein amino acid sequence. Starting from a randomly distributed gas of DRs inside a spherical volume of diameter *D*_*max*_, a simulated annealing routine was employed to find a chain-compatible spatial distribution of DRs which fit the experimental scattering pattern. Ten different calculations were also performed and the NSD values were evaluated. In this case, there is no advantage in obtaining an average model because the GASBOR program uses a predefined number of DRs, which makes the average routine little effective in achieving an improvement of the model resolution. So, we present the most typical model (with the lowest NSD value). In both approaches, the models calculated with 2 point-symmetry constraint were very similar to those calculated without these constraints. For this reason, the results presented here are from the calculation with 2 point-symmetry constraint. Both models were displayed by the PyMOL program [80].

## Authors' contributions

DMT and JK conceived and designed the experiments, analyzed the data and wrote the manuscript. DMT performed the experiments. JCS performed SAXS experiments and interpreted them together with ICLT. MSN performed and interpreted the mass spectrometry experiments. JK supervised the project. All authors read and approved the final version of the manuscript.

## Supplementary Material

Additional file 1 **Original UPLC-ESI-QTOF and MALDI-QTOF data (a)**. Spectra of the trypsin data presented in Table 2 (part a) Click here for file

Additional file 2 **Original UPLC-ESI-QTOF and MALDI-QTOF data (b)**. Spectra of the chymotrypsin data presented in Table 2 (part b) Click here for file

Additional file 3 **Original UPLC-ESI-QTOF and MALDI-QTOF data (c)**. Spectra of the formic acid data presented in Table 2 (part c) Click here for file
