# Supplementary material for: Low-resolution structural studies of human Stanniocalcin-1
Source: BMC Struct Biol. 2009 Aug 27;9:57. doi: 10.1186/1472-6807-9-57 (PMC2744999; doi:10.1186/1472-6807-9-57)
Supplement: Additional file 1 — Original UPLC-ESI-QTOF and MALDI-QTOF data (a). Spectra of the trypsin data presented in Table 2 (part a) [file 1472-6807-9-57-S1.ppt]

## Slide 1
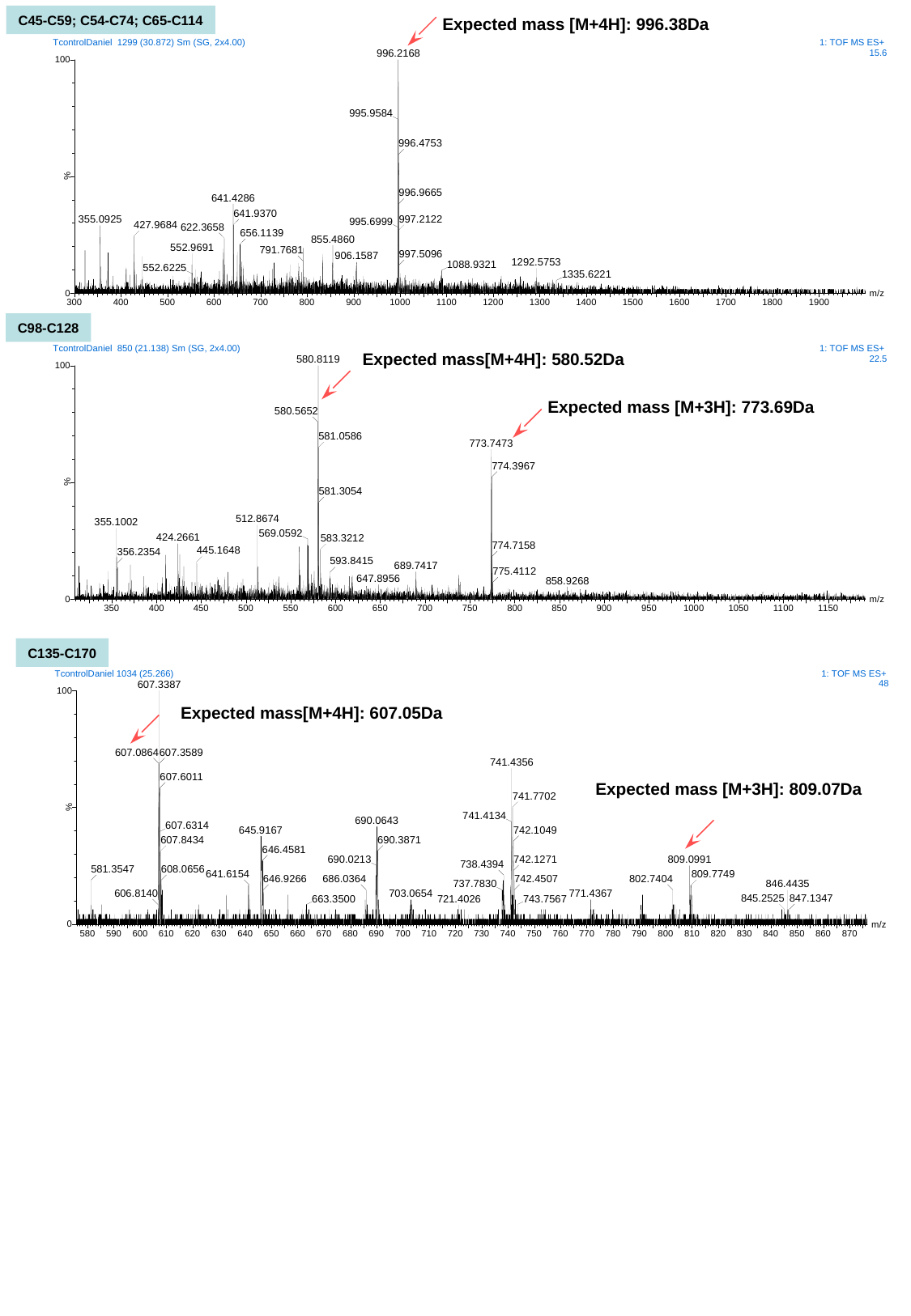

C45-C59; C54-C74; C65-C114
Expected mass [M+4H]: 996.38Da
C98-C128
Expected mass[M+4H]: 580.52Da
Expected mass [M+3H]: 773.69Da
C135-C170
Expected mass[M+4H]: 607.05Da
Expected mass [M+3H]: 809.07Da
