# Supplementary material for: Low-resolution structural studies of human Stanniocalcin-1
Source: BMC Struct Biol. 2009 Aug 27;9:57. doi: 10.1186/1472-6807-9-57 (PMC2744999; doi:10.1186/1472-6807-9-57)
Supplement: Additional file 2 — Original UPLC-ESI-QTOF and MALDI-QTOF data (b). Spectra of the chymotrypsin data presented in Table 2 (part b) [file 1472-6807-9-57-S2.ppt]

## Slide 1
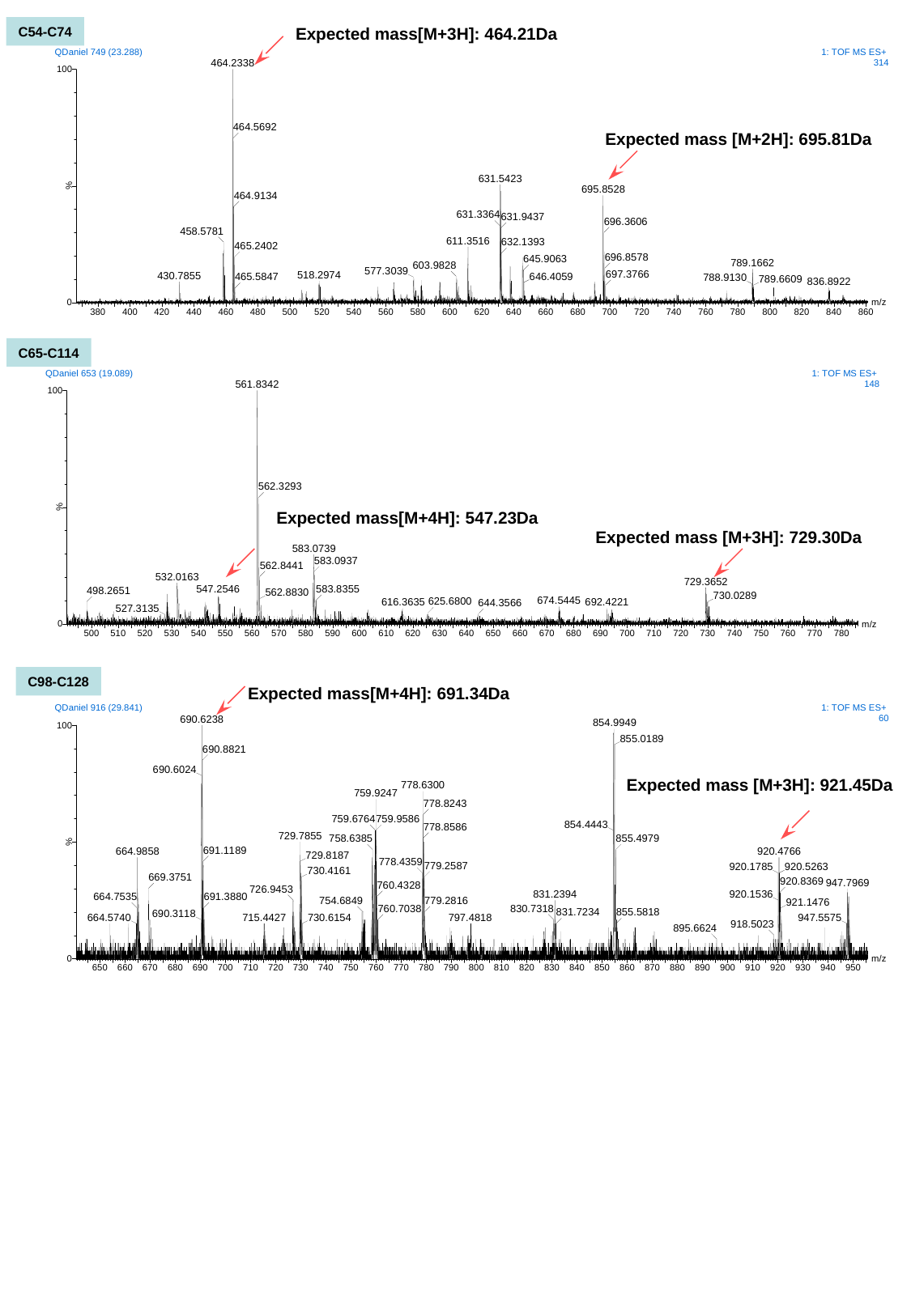

C54-C74
Expected mass[M+3H]: 464.21Da
Expected mass [M+2H]: 695.81Da
C65-C114
Expected mass[M+4H]: 547.23Da
Expected mass [M+3H]: 729.30Da
C98-C128
Expected mass[M+4H]: 691.34Da
Expected mass [M+3H]: 921.45Da
