# Supplementary figures and images for: Low-resolution structural studies of human Stanniocalcin-1
Source: BMC Struct Biol. 2009 Aug 27;9:57. doi: 10.1186/1472-6807-9-57 (PMC2744999; doi:10.1186/1472-6807-9-57)

## Slide 1
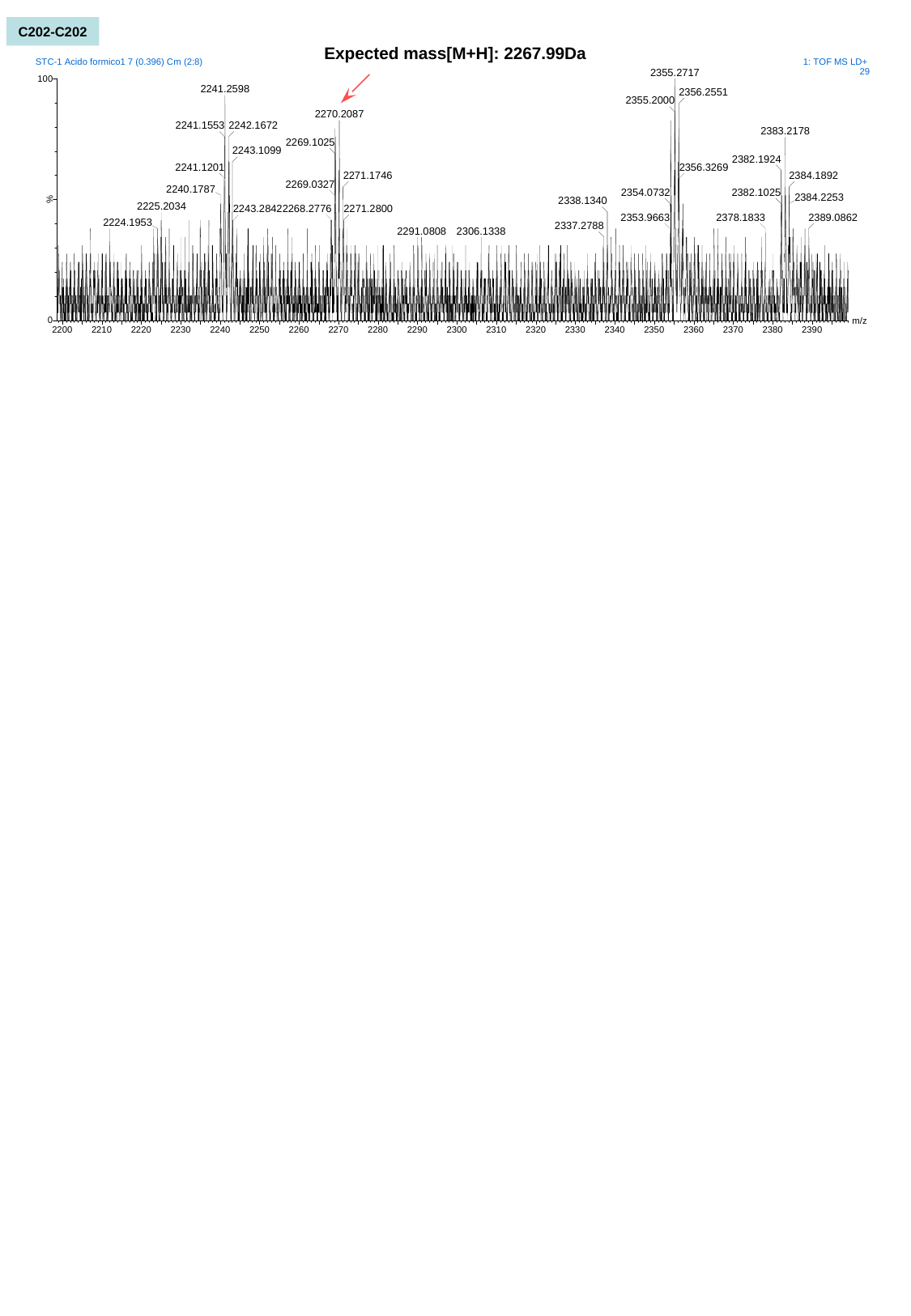

C202-C202
Expected mass[M+H]: 2267.99Da

Supplement: Additional file 3 — Original UPLC-ESI-QTOF and MALDI-QTOF data (c). Spectra of the formic acid data presented in Table 2 (part c) [file 1472-6807-9-57-S3.ppt]
